# The Fast Territorial Expansion of COVID-19 in Spain

**DOI:** 10.2188/jea.JE20200123

**Published:** 2020-05-05

**Authors:** Marcelino Perez-Bermejo, Maria Teresa Murillo-Llorente

**Affiliations:** SONEV Research Group, School of Medicine and Health Sciences, Catholic University of Valencia San Vicente Martir, Valencia, Spain

## THE FAST TERRITORIAL EXPANSION OF COVID-19 IN SPAIN

During the second week of isolation decreed by the Spanish Government, there has been an increase in the population infected with the COVID-19 coronavirus that no expert had predicted. In Spain, deaths have doubled in just 3 days, which is a higher rate than that of the two countries most affected by the coronavirus, China and Italy.

On the other hand, territorial expansion has been much greater in Spain than in Italy. In Italy, the main source of contagion was located in the Lombardy, Emilia-Romagna and Veneto regions, located in the north of the country. However, in Spain, there has been a much greater increase throughout the country.

Chinese authorities introduced unprecedented measures to contain the virus, dramatically halting the movement of more than 60 million people, forcing people in many Chinese cities to stay home and go out alone to find food or medical help, leaving approximately 760 million people confined to their homes. The World Health Organization congratulated the Chinese government on these measures.

The subsequent evolution of the cases in China seems to indicate that the drastic measures had the desired effect, despite the opinions of scientists who believed that many cases were not reported because they had not been diagnosed or that China’s response started too late.

But one of the key points in developing China’s response to the threat was the suspension of public transportation within the city, closing entertainment venues and banning public gatherings. According to a preprint^1^ that analyzes the impact of transmission control measures during the first 50 days of the COVID-19 epidemic in China, cities that implemented these measures had 33.3% fewer cases than cities that did not implement them.

What really matters in this situation is not so much the analysis of the effects of the measures taken in China, but rather looking for what is the difference of the measures taken in Spain with respect to those, in order to analyze why territorial transmission has been much faster in Spain. Possible responses can help countries where the infection is still in its infancy not to make the same mistakes.

A priori, one of the biggest differences has been the government response. Even knowing the world evolution of the pandemic, mobility in Spain remained unchanged until the declaration of the State of Alarm and the entry of citizens from countries severely affected by the pandemic, especially from Italy, was allowed without any restrictions. In addition, the celebration of numerous mass demonstrations was authorized on March 8, on the occasion of Women’s Day, mainly in the city of Madrid, as well as many mass celebrations in the Fallas of Valencia and sports celebrations, which may partially explain the enormous impact that the infection is having in the Capital of Spain, whose figures far exceed those of other cities 14 days after these celebrations (the incubation time for the virus).

The accumulated experience and the rapid territorial expansion by all the communities of Spain can be used to advise countries with incipient infections on the benefits of rapid and drastic application of measures to stop this expansion, in addition to all the recommendations of the health authorities to avoid infections. The sad experience suffered in Spain encourages us to communicate this to the rest of the world, so that, as far as possible, they can take measures sufficiently in advance to avoid rapid territorial expansion.
